# Effects of Emotional Valence and Concreteness on Children’s Recognition Memory

**DOI:** 10.3389/fpsyg.2020.615041

**Published:** 2020-12-04

**Authors:** Julia M. Kim, David M. Sidhu, Penny M. Pexman

**Affiliations:** Department of Psychology, University of Calgary, Calgary, AB, Canada

**Keywords:** lexical knowledge, concreteness, recognition memory, affective embodiment, word valence

## Abstract

There are considerable gaps in our knowledge of how children develop abstract language. In this paper, we tested the Affective Embodiment Account, which proposes that emotional information is more essential for abstract than concrete conceptual development. We tested the recognition memory of 7- and 8-year-old children, as well as a group of adults, for abstract and concrete words which differed categorically in valence (negative, neutral, and positive). Word valence significantly interacted with concreteness in hit rates of both children and adults, such that effects of valence were only found in memory for abstract words. The pattern of valence effects differed for children and adults: children remembered negative words more accurately than neutral and positive words (a negativity effect), whereas adults remembered negative and positive words more accurately than neutral words (a negativity effect and a positivity effect). In addition, signal detection analysis revealed that children were better able to discriminate negative than positive words, regardless of concreteness. The findings suggest that the memory accuracy of 7- and 8-year-old children is influenced by emotional information, particularly for abstract words. The results are in agreement with the Affective Embodiment Account and with multimodal accounts of children’s lexical development.

## Introduction

Abstract words refer to concepts that are difficult to experience through the senses, like *truth*, *love*, and *think*. Between the ages of 4 and 12, a child’s abstract vocabulary grows from less than 10% of words known to more than 40%, with the rate of acquisition peaking between ages seven and eight (Ponari et al., 2018). Very little research has considered how children acquire and represent the meanings of abstract words. The goal of the present study was to address this question.

It has been a challenge for some theories of lexical and conceptual knowledge to explain the acquisition of abstract concepts. There are several different theoretical views on how lexical knowledge is represented, and they can be placed on a spectrum based on the role of sensory and motor systems (Meteyard et al., 2012). On the one hand, amodal accounts hold that knowledge is represented symbolically and separately from other systems (e.g., Quillian, 1969; Pylyshyn, 1985). On the other hand, strongly embodied accounts assume that all knowledge is grounded in sensory, motor, and emotion systems (e.g., Glenberg and Gallese, 2012; Glenberg, 2015). In between lie multimodal accounts, which suggest that knowledge is represented in language, emotion, perceptual, sensory, and motor systems, and that the influence of each system depends on the types of concepts being processed (e.g., Barsalou et al., 2008; Borghi et al., 2017).

The strongly embodied accounts have the greatest difficulty explaining acquisition of abstract vocabulary; if word meanings cannot be experienced through the senses, how are they acquired? Amodal or unembodied accounts explain acquisition of abstract meaning in terms of linguistic representation: the meanings of abstract words are represented by association with already learned words (Howell et al., 2005). Multimodal accounts suggest that there are multiple avenues through which word meaning is acquired, including emotional, sensorimotor, and linguistic information associated with the referent (e.g., Kousta et al., 2009; D’Angiulli et al., 2015; Moffat et al., 2015; Xue et al., 2015). For instance, Vigliocco et al. (2009) proposed that emotional knowledge forms the basis for many abstract concepts, whereas sensorimotor knowledge forms the basis for concrete ones.

Within the multimodal framework, it has been suggested that the emotional information associated with a word (i.e., word valence), provides a bootstrapping mechanism for children’s abstract vocabulary acquisition (Kousta et al., 2011). More specifically, the meanings of children’s first abstract words may be grounded in felt experience, such as associating the label “love” with the feeling of being hugged. Indeed, Kousta et al. (2011) showed that abstract words acquired earlier tended to be more emotionally valenced. Borghi et al. (2017) proposed the Affective Embodiment Account, by which emotion information is more essential to learning of abstract concepts, while sensorimotor experience is more vital to learning of concrete concepts. The account thus predicts that emotion information should be more important to the acquisition and representation of abstract concepts than concrete concepts.

In adults, word valence affects lexical processing speed, with positive words processed faster than neutral words. Negative words are sometimes processed more slowly than neutral words (Estes and Adelman, 2008) and sometimes more quickly (Kousta et al., 2009; Vinson et al., 2014). Slower lexical processing for negative words is attributed to automatic vigilance for negative information (Pratto and John, 1991), which delays lexical access. Faster lexical processing for negative words, as for positive words, is attributed to semantic richness (Siakaluk et al., 2016). In line with the Affective Embodiment Account, there is some evidence from adult lexical processing studies that emotion has a stronger influence on processing of abstract words than concrete words, in tasks such as word naming and semantic categorization (Newcombe et al., 2012; Moffat et al., 2015).

Two previous developmental studies have found some support for predictions of the Affective Embodiment Account by testing the effects of word valence and concreteness on children’s lexical processing (Ponari et al., 2018; Lund et al., 2019). Both of these studies employed an auditory lexical decision task (ALDT; *is it a real word?*) to tap children’s word representations, and both reported challenges with measuring children’s performance in this task. Ponari et al. (2018) included a limited set of stimuli and measured ALDT response accuracy in 6- to 12-year-old children. They found that only 8- to 9-year-olds showed an effect of valence (more accurate responses for positive words than neutral words), and that this was limited to abstract words; there was no valence effect for concrete words. Ponari et al. (2018) also found that the youngest children they tested (6- to 7-year-olds) essentially performed at chance in the task. They noted that it would have been challenging to measure and analyze children’s reaction times in the ALDT in light of all potential confounds that can influence auditory word processing time (e.g., first phoneme, uniqueness point; Goldinger, 1996). Lund et al. (2019) used a larger set of items in their study with 5- to 7-year-old children and analyzed reaction time. They found an effect of valence (faster responses for positive words than negative words) that was limited to more abstract words. Lund et al. (2019) included predictors in their analysis to account for many potential confounds, but found limits to the inferences that could be drawn from reaction time data. For instance, the oldest children they tested (7-year-olds) responded especially quickly which may have limited the effects observed.

To further test predictions of the Affective Embodiment Account, in the present study, we shifted focus to a different cognitive process and investigated the effects of word valence and concreteness on children’s recognition memory. This allowed us to test whether children’s retrieval of abstract and concrete words are differentially influenced by emotional information. To our knowledge, the relationship between concreteness and emotional enhancement of memory has not yet been investigated, in either children or adults.

Emotional enhancement of memory is a phenomenon in which positive and negative emotional information is better remembered than neutral information. It has been demonstrated in adult studies using various stimuli, including pictures, stories, and words (LaBar and Cabeza, 2006; Kensinger and Schacter, 2008; Adelman and Estes, 2013). Some studies have suggested that the enhancement is actually comprised of two effects: *the positivity effect*, involving better memory for positive information (vs. neutral), and *the negativity effect*, involving better memory for negative information (vs. neutral; Murphy and Isaacowitz, 2008). In a meta-analysis by Murphy and Isaacowitz (2008), younger adults tended to show a negativity effect, whereas older adults were more likely to show a positivity effect. Other studies have shown that younger children exhibit an attentional bias to negatively valenced stimuli (for a review, see Morales et al., 2016). Thus, the negativity effect in memory is attributed to an attentional bias to negative stimuli, which is thought to be present in childhood, whereas the positivity effect in memory is attributed to later-developing emotion regulation skills (Murphy and Isaacowitz, 2008). The nature of the emotional enhancement effect, therefore, seems to change with development, likely due to age-related differences in emotional processing and regulation (Michalska and Davis, 2019).

Many developmental studies have examined the effects of valence on memory using images or stories as stimuli (Davidson et al., 2001; Prehn-Kristensen et al., 2009; Cordon et al., 2013; Leventon et al., 2014; Van Bergen et al., 2015). This is in contrast with studies on adults. Indeed, in their review of emotional memory research, Hamann and Stevens (2014) noted that developmental studies have tended to use methods and materials that are different than those used in adult studies, making comparisons challenging. Only a handful of studies have examined the effects of emotion on children’s memory using word stimuli. Quas et al. (2016) showed no effect of valence on recognition memory in children aged seven and eight. Adolescents, however, who were 12–14 years of age, showed significantly better memory for negative and positive words compared to neutral words. In contrast, Howe et al. (2010) found that 7- and 11-year-old children had more accurate recognition memory for neutral words than for negative words (positive words were not included), and that adults showed no significant differences. Notably, the word lists used by Quas et al. (2016) and Howe et al. (2010) were devised for the standard false memory paradigm, and therefore the words within each list were semantically related and of the same valence. As such, list context might have influenced memory for individual word stimuli.

Recently, Massol et al. (2020) tested the memory of adults and 9- to 11-year-old children for stimuli that included unrelated negative, neutral, and positive words. Participants completed a recognition task followed by a free-recall task. Adults and children recalled positive and negative words more accurately than neutral words. In the recognition memory task, however, there were no significant effects of valence.

In short, there is limited research on the effects of word valence on children’s memory. Moreover, the existing research has used varying and inconsistent methods with mixed results, which calls for additional exploration. In the present study, we examined the effects of word valence and concreteness on adults’ and children’s recognition memory. We tested 7- and 8-year-old children, since this is the age of rapid abstract word acquisition, and compared the groups’ performance to each other. Since children have greater neural response and attentional bias toward negative stimuli (Leventon et al., 2014; Morales et al., 2016), we hypothesized that children might show a negativity effect in memory accuracy, with better recognition memory for negative than positive and neutral words. No previous studies have examined the interaction of word valence and concreteness in recognition memory, but based on the Affective Embodiment Account, we hypothesized greater emotional enhancement of memory for abstract words compared to concrete words.

## Materials and Methods

### Participants

The child participants were 45 7-year-olds (*M* = 7.50 years, *SD* = 0.27, 21 female) and 42 8-year-olds (*M* = 8.50 years, *SD* = 0.28, 23 female). All children were native English speakers, with 22 bilingual or multilingual children, whose parents indicated that their everyday usage of languages other than English was less than 30%. In addition, 42 adults (*M* = 19.55 years, *SD* = 3.24, range = 17–34 years, 32 female) were recruited through the university’s research participation system. This study was approved by the University of Calgary Conjoint Faculties Research Ethics Board. Informed consent was obtained from the child participants’ parents and from adult participants, and verbal assent was obtained from the children before beginning the tasks.

### Stimuli

We selected 120 monosyllabic words to achieve a factorial manipulation of valence (negative, positive, neutral) and concreteness (abstract, concrete). The negative words had valence ratings of 1–4, neutral words had valence ratings of 4.01–6.50, and the positive words had valence ratings of 6.51–9, based on the ratings from Warriner et al. (2013). Each valence group was further divided into abstract and concrete groups using a median split based on the concreteness ratings of Brysbaert et al. (2014). Thus, six groups of 20 items were created (Table 1). The word groups were matched on several variables that are important in lexical processing and recognition memory: phonological Levenshtein distance (PLD, Sanders and Chin, 2009), children’s spoken frequency for 84–95 months (ChildFreq norms, Bääth, 2010), Grade 2 print frequency (Zeno et al., 1995), age of acquisition (Kuperman et al., 2012), imageability (Cortese and Fugett, 2004), and word length. The frequency and age of acquisition values for these words suggested that they should be familiar to children in the age range we tested. See Table 1. We also selected 120 monosyllabic non-words from the English Lexicon Project (Balota et al., 2007) for the encoding task. These were matched to the real words on number of phonemes and phoneme onsets.

**TABLE 1:** Mean characteristics of word stimuli for each word type (standard deviations in parentheses).

	Word type							
	Negative		Neutral		Positive		*p* for variable effect	
	Abstract	Concrete	Abstract	Concrete	Abstract	Concrete	Valence	Concreteness
	**Word characteristics**							
Valence	2.95 (0.60)	3.31 (0.50)	5.53 (0.60)	5.38 (0.62)	7.07 (0.50)	7.04 (0.44)	<0.001	0.71
PLD	1.23 (0.29)	1.42 (0.30)	1.26 (0.33)	1.39 (0.30)	1.42 (0.37)	1.24 (0.25)	1	0.34
Child spoken frequency	47.95 (86.65)	17.05 (37.06)	44.70 (152.53)	38.55 (146.50)	26.16 (51.13)	44.67 (174.59)	0.94	0.82
Grade 2 print frequency	34.95 (56.75)	28.70 (29.34)	26.15 (19.80)	54.4 (155.37)	54.47 (107.98)	34.81 (43.42)	0.80	0.90
Age of acquisition	6.91 (1.83)	6.83 (1.36)	6.69 (1.31)	7.08 (1.45)	6.85 (1.27)	6.70 (1.15)	0.93	0.80
Imageability	3.52 (0.77)	5.29 (0.78)	3.52 (1.00)	5.33 (0.75)	3.40 (0.55)	5.39 (0.71)	0.98	<0.001
Concreteness	2.82 (0.64)	4.29 (0.37)	2.85 (0.41)	4.26 (0.48)	2.61 (0.58)	4.36 (0.40)	0.96	<0.001
Length	4.30 (0.92)	4.55 (0.83)	4.45 (0.89)	4.40 (0.68)	4.47 (1.07)	4.38 (0.97)	1	0.92

*PLD, phonological Levenshtein distance.*

For the recognition memory (retrieval) task, an additional 40 positive, 40 neutral, and 40 negative monosyllabic words were selected as foils. These were matched to the original words on valence, age of acquisition, and length in letters. All stimuli are available at https://osf.io/69syt/. All of the word stimuli were recorded by a female speaker using Audacity v. 2.3.0 (Mazzoni and Dannenberg, 2000). The speaker was unaware of the study purpose to prevent unintended manipulation of tone. Sound recordings were edited using Audacity (Mazzoni and Dannenberg, 2000) and Praat (Boersma and Weenink, 2018) to remove ambient sounds. Duration of word recordings did not significantly differ across each valence group. Word recordings were evaluated by three adult listeners. Some words were re-recorded based on the feedback provided by the listeners, until all could be accurately recognized. Table 1.

### Procedure

Procedures for child and adult participants were similar, except for minor differences in the filler and language tasks. The study consisted of encoding, filler, and retrieval phases.

During the encoding phase, participants were presented with 10 practice trials followed by 120 words and 120 non-words. Participants were not told that their memory for the words would be tested in the subsequent retrieval session. Participants sat beside the experimenter, in front of a computer screen. Both wore headphones. Participants were instructed to distinguish real and fake words as if they were “word detectives” via button press. On each trial, a white fixation cross was presented on a black background. An audio recording was presented via headphones. After the participant pressed one of the two buttons on the response box, the fixation cross was replaced with a letter “B,” and the experimenter pressed a key to proceed to the next trial. The order of word presentation was randomized and stimuli were presented using E-prime (Schneider et al., 2001).

After the encoding phase, participants completed the filler task at an adjacent table, consisting of five manual dexterity subscales of the Bruininks-Oseretsky Test of Motor Proficiency (BOT-2; Bruininks and Bruininks, 2005). Child participants completed all five tasks, whereas adults completed the first four, with the fifth task replaced by an imagery task of the same length. Administration of the BOT ensured a consistent 10-min interval between encoding and the recognition memory task. Children’s mean BOT score was 25.82 (*SD* = 3.33).

In the retrieval phase, participants returned to the computer and completed the recognition memory task. They were told that they were playing a memory game and were asked to distinguish words heard in the encoding task (i.e., old) from words they did not hear in the encoding task (i.e., new) via button press. We presented 240 trials, in a random order, including 120 old and 120 new words.

For child participants, the Peabody Picture Vocabulary Test (PPVT-4; Dunn and Dunn, 2007), was administered. In addition, children’s parents completed the Children’s Communication Checklist-2 (CCC-2; Bishop, 2006). Children’s mean scores for PPVT and CCC-2 were 148.30 (*SD* = 16.17) and 82.13 (*SD* = 13.75), respectively, suggesting the sample was within normal limits for language development.

## Results

One common way of analyzing recognition memory data is to use a signal detection paradigm (Tajika, 2001; Dunn, 2010). This paradigm models participants’ decisions regarding whether an item comes from a target distribution (i.e., previously seen, or *old*, items) as compared to a distractor distribution (i.e., lure, or *new*, items), based on the memory strength of a given item. This involves calculating each participant’s *hit rate* (i.e., the percentage of correctly identified old items) and *false alarm rate* (i.e., the percentage of incorrectly identified new items). These are then used to calculate each participant’s *d’ score*, which represents their ability to discriminate old from new items. d’ is calculated by subtracting a participant’s standardized false alarm rate (using the inverse of the Standard Normal Cumulative Distribution) from their standardized hit rate (higher values reflect a better ability to discriminate; Macmillan and Creelman, 1991). A final measure is *Criterion C*, which quantifies the willingness of a participant to label an item as old. It is calculated by summing a participant’s standardized hit rate and false alarm rate, and multiplying the sum by −0.5 (lower values reflect a more liberal response criterion; Macmillan and Creelman, 1991). All data for the present study are available at https://osf.io/69syt/.

### Children’s Responses

**FIGURE 1:**
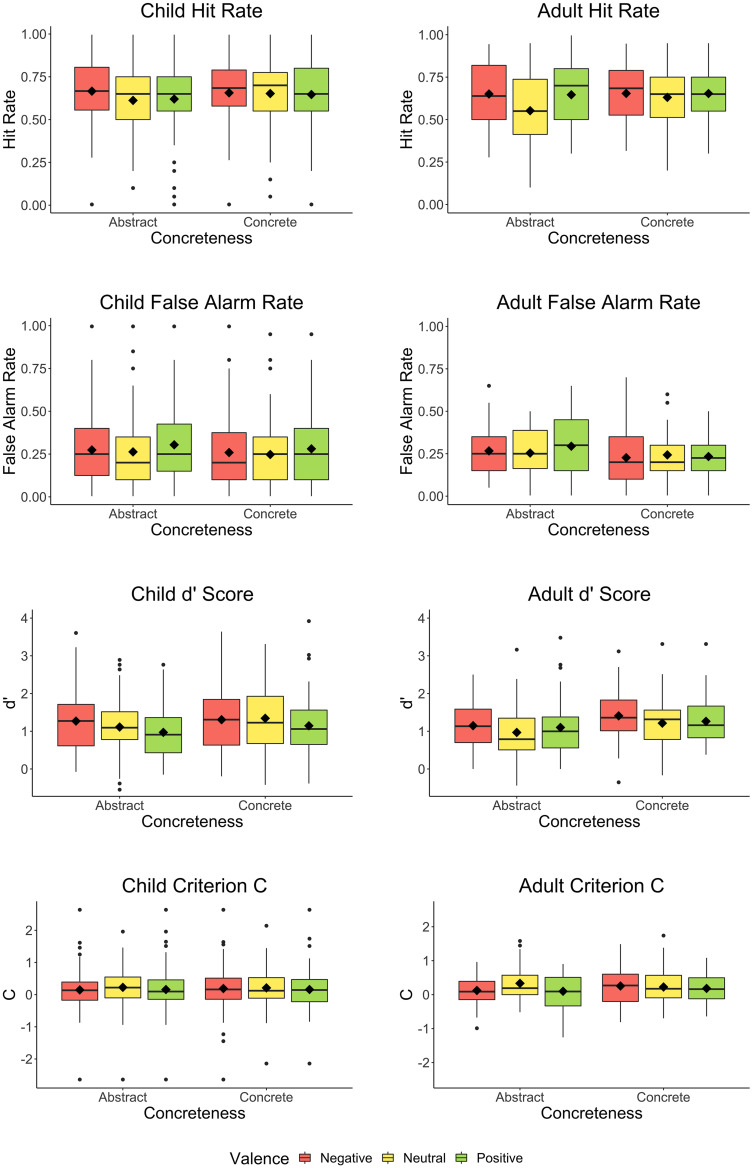
Children’s and adults’ hit rates, false alarm rates, d’ scores, and Criterion C values for words as a function of valence and concreteness. Horizontal bars indicate medians; diamonds indicate means. Colored boxes correspond to the range between the first and third quartiles (i.e., the 25th and 75th percentiles). Whiskers indicate the lowest and highest values up to 1.5 times the interquartile range from the first and third quartiles, respectively. Dots represent all other values beyond the whiskers.

We removed three words that had accuracy <50 in the encoding task: *tramp*, *sly*, and *sue*. For each of the six types of words we calculated each participant’s hit rate, false alarm rate, d’ score, and Criterion C. When hit rate or false alarm rate was equal to zero or one, we used the correction suggested by Macmillan and Creelman (1991). As is typical in the recognition memory literature, analyses were 3 (valence: neutral vs. negative vs. positive) × 2 (concreteness: abstract vs. concrete) × 2 (age: 7- vs. 8-year-old) ANOVAs. This analysis was run on four separate dependent variables: hits, false alarms, d’ score, and Criterion C. When sphericity was violated we report results using the Greenhouse–Geisser correction. We used the package “afex” (Singmann et al., 2020) to compute ANOVAs, “groupedstats” (Patil, 2018) to calculate partial eta-squared values, and “effectsize” (Ben-Shachar et al., 2020) to calculate Cohen’s *d* values. Analyses were conducted in R (4.0.3) (R Core Team, 2018). Code for all analyses is available here: https://osf.io/69syt/. Results are illustrated in Figure 1.

#### Hits

The three-way interaction was not significant [*F*(1.90, 161.87) = 1.08, *p* = 0.34, η^2^*p* = 0.01]. However, there was a significant interaction between valence and concreteness [*F*(1.90, 161.87) = 3.42, *p* = 0.037, η^2^*p* = 0.04]. We followed up this interaction with one-way ANOVAs with valence as a predictor, on abstract and concrete words separately. For abstract words there was a significant effect of valence [*F*(1.93, 165.82) = 6.70, *p* = 0.002, η^2^*p* = 0.07]. We investigated effects of valence in abstract items using Bonferroni corrected *t*-tests (α = 0.017) and found negative words had a significantly higher hit rate (*M* = 0.67, *SD* = 0.19) than neutral words (*M* = 0.61, *SD* = 0.19, *p* = 0.001, Cohen’s *d* = 0.38^1^); the hit rate for positive words (*M* = 0.62, *SD* = 0.19) did not significantly differ from neutral words (*p* = 0.59, Cohen’s *d* = 0.06). Negative words also had a significantly higher hit rate than positive words (*p* = 0.01, Cohen’s *d* = 0.28). For concrete words there was no effect of valence [*F*(1.87, 161.13) = 0.22, *p* = 0.79, η^2^*p* = 0.00]. No other two-way interactions reached significance (*p*s > 0.08). There was a significant main effect of age [*F*(1, 85) = 4.07, *p* = 0.047, η^2^*p* = 0.05], such that 7-year-olds had a significantly lower hit rate (*M* = 0.61, *SD* = 0.20) than 8-year-olds (*M* = 0.68, *SD* = 0.16).

#### False Alarms

No interactions reached statistical significance (*p*s > 0.58). There were significant effects of valence [*F*(1.90, 161.35) = 9.15, *p* < 0.001, η^2^*p* = 0.10] and concreteness [*F*(1, 85) = 5.02, *p* = 0.028, η^2^*p* = 0.06]. We investigated effects of valence using Bonferroni corrected *t*-tests (α = 0.017) and found positive words had a significantly higher false alarm rate (*M* = 0.29, *SD* = 0.20) than neutral words (*M* = 0.26, *SD* = 0.19, *p* < 0.001, Cohen’s *d* = 0.43); negative words (*M* = 0.27, *SD* = 0.20) did not significantly differ from neutral words (*p* = 0.15, Cohen’s *d* = 0.16). Negative words had a significantly lower false alarm rate than positive words (*p* = 0.008, Cohen’s *d* = 0.29). The effect of concreteness was such that abstract words (*M* = 0.28, *SD* = 0.19) had a higher false alarm rate than concrete words (*M* = 0.26, *SD* = 0.19). There was no main effect of age [*F*(1, 85) = 0.02, *p* < 0.88, η^2^*p* = 0.00].

#### d’ Score

No interactions reached statistical significance (*p*s > 0.24). There were significant effects of valence [*F*(1.89, 160.48) = 8.72, *p* < 0.001, η^2^*p* = 0.09] and concreteness [*F*(1, 85) = 10.01, *p* = 0.002, η^2^*p* = 0.11]. We investigated effects of valence using Bonferroni corrected *t*-tests (α = 0.017) and found that positive words had a significantly lower d’ score (*M* = 1.06, *SD* = 0.60) than neutral words (*M* = 1.23, *SD* = 0.70, *p* = 0.001, Cohen’s *d* = 0.37); negative words (*M* = 1.29, *SD* = 0.75) did not significantly differ from neutral words (*p* = 0.31, Cohen’s *d* = 0.11). Negative words had a significantly higher d’ than positive words (*p* < 0.001, Cohen’s *d* = 0.40). The effect of concreteness was that concrete words (*M* = 1.27, *SD* = 0.71) had a higher d’ score than abstract words (*M* = 1.12, *SD* = 0.59). There was no main effect of age [*F*(1, 85) = 1.88, *p* = 0.17, η^2^*p* = 0.02].

#### Criterion C

No interactions reached statistical significance (*p*s > 0.09), nor did any main effects (*p*s > 0.066).

### Adults’ Responses

To facilitate comparison with children’s responses, we removed the same three items that were removed from those analyses. Data preparation and analyses were the same as in the child analyses, but without age as a factor.

#### Hits

The analysis revealed a significant interaction of valence and concreteness [*F*(1.97, 80.92) = 3.41, *p* = 0.038, η^2^*p* = 0.08]. We followed up this interaction with one-way ANOVAs with valence as a predictor, on abstract and concrete words separately. The analysis of abstract words revealed a significant effect of valence [*F*(1.82, 74.76) = 8.74, *p* < 0.001, η^2^*p* = 0.18]. We investigated effects of valence for abstract items using Bonferroni corrected *t*-tests (α = 0.017) and found that positive words had a significantly higher hit rate (*M* = 0.65, *SD* = 0.19) than neutral words (*M* = 0.55, *SD* = 0.21, *p* < 0.001, Cohen’s *d* = 0.66), as did negative words (*M* = 0.65, *SD* = 0.18, *p* = 0.001, Cohen’s *d* = 0.55). Negative and positive words did not differ significantly (*p* = 0.88, Cohen’s *d* = 0.02). For concrete words there was no effect of valence [*F*(1.89, 77.39) = 0.64, *p* = 0.52].

#### False Alarms

The interaction between valence and concreteness was not significant [*F*(1.97, 80.78) = 1.50, *p* = 0.23, η^2^*p* = 0.04], nor was the main effect of valence [*F*(1.91, 78.17) = 0.71, *p* = 0.49, η^2^*p* = 0.02]. There was a significant effect of concreteness [*F*(1, 41) = 13.16, *p* = 0.001, η^2^*p* = 0.24] such that abstract words had a higher false alarm rate (*M* = 0.27, *SD* = 0.14) than concrete words (*M* = 0.23, *SD* = 0.12).

#### d’ Score

The interaction between valence and concreteness was not significant [*F*(1.94, 79.45) = 0.28, *p* = 0.75, η^2^*p* = 0.01], nor was the main effect of valence [*F*(2, 81.98) = 2.18, *p* = 0.12, η^2^*p* = 0.05]. There was a significant effect of concreteness [*F*(1, 41) = 10.91, *p* = 0.002, η^2^*p* = 0.21] such that concrete words had a higher d’ score (*M* = 1.29, *SD* = 0.53) than abstract words (*M* = 1.07, *SD* = 0.58).

#### Criterion C

The analysis revealed a significant interaction [*F*(1.81, 74.28) = 4.56, *p* = 0.016, η^2^*p* = 0.10]. We followed up this interaction with one-way ANOVAs with valence as a predictor, on abstract and concrete words separately. For abstract words there was a significant effect of valence [*F*(1.87, 76.86) = 8.03, *p* < 0.001, η^2^*p* = 0.16]. We investigated effects of valence in abstract items using Bonferroni corrected *t*-tests (α = 0.017) and found that positive words had a significantly lower Criterion C (*M* = 0.10, *SD* = 0.50) than neutral words (*M* = 0.33, *SD* = 0.54, *p* < 0.001, Cohen’s *d* = 0.66), as did negative words (*M* = 0.12, *SD* = 0.43, *p* = 0.004, Cohen’s *d* = 0.48). Negative and positive words did not differ significantly (*p* = 0.73, Cohen’s *d* = 0.05). For concrete words there was no effect of valence [*F*(1.73, 70.94) = 0.60, *p* = 0.53, η^2^*p* = 0.01].

## Discussion

The purpose of the present study was to investigate emotional enhancement of memory and its differential effect for abstract and concrete words, in a developmental context. We tested predictions derived from the Affective Embodiment Account, that emotional enhancement might be stronger for abstract words than for concrete words.

We measured several aspects of children’s and adults’ memory performance. One of these provided support for the Affective Embodiment Account: we found interactions of valence and concreteness in both children’s and adults’ hit rates (correct identification of “old” items), such that effects of valence were significant for abstract but not for concrete word memory. This supported the assumptions that for abstract words emotional information is important for grounding word meanings, and that for concrete words, which have more grounding in sensorimotor networks, valence is less influential.

We presented child and adult participants with the same stimuli and found both similarities and differences in memory effects. In particular, the nature of the valence effects for abstract word memory differed for children and adults. While children showed more accurate hit rates for negative (vs. neutral and positive) abstract words, adults showed higher hit rates for negative *and* positive (vs. neutral) abstract words. For adults, the interaction was also observed for Criterion C, such that they were particularly conservative (unlikely to say “old”) in their responses for neutral abstract words. This suggests that for adults, valence influences both their memory for the words (hit rates) and their decision strategy (Criterion C).

Adults’ valence effects showed the usual *u*-shaped curve (Adelman and Estes, 2013), with better memory for valenced words (both positive and negative) than for neutral words. Children’s valence effects took a different form, consistent with the hypothesis that children might be more likely to show a negativity than a positivity effect. The negativity effect we observed for children is in keeping with findings from the previous literature which suggest that children have a greater neuronal response and attentional bias for negative stimuli than for positive stimuli (Leventon et al., 2014; Morales et al., 2016). One potential explanation for the negativity bias could be its evolutionary function, as avoidance of harmful stimuli helps increase chances for survival (Vaish et al., 2008).

The differences in the nature of the valence effects we observed for children and adults may be due to developmental differences in emotional processing. Children undergo significant development in their ability to understand, regulate, and change emotional responses (for a review, see Michalska and Davis, 2019). It could be the case that as children develop stronger emotion regulation skills, they begin to show effects of positive valence on memory accuracy, similar to adults. This research question should be addressed in future studies with older children.

In contrast to the results described for hits, our other measures of children’s memory performance showed main effects of valence and concreteness separately, without an interaction. Specifically, children showed better discrimination in their memory for negative than for positive words. They also made more false alarms (judging that new items were “old”) for positive words than for negative and neutral words. When words were of positive valence, children were more likely to incorrectly respond that they had seen those words before. Our findings are reasonably consistent with previous observations of valence effects in children’s word recall (Howe et al., 2010; Massol et al., 2020), even with different stimuli and with a different retrieval task. More broadly, our results are consistent with Leventon et al.’s (2014) assertion that emotion effects can be detected in children’s memory performance at 7–8 years of age. Whereas Leventon et al. (2014) found significant effects of valence on children’s ERP response to visual scenes, we found them on children’s memory for words.

For both children and adults, word concreteness influenced memory performance. Both groups produced more false alarms for abstract than for concrete words, and they also showed more accurate discrimination for concrete than abstract words. This recognition memory advantage for concrete words is consistent with previous literature (e.g., Taylor et al., 2019). There may be more retrieval pathways for concrete than abstract words, by virtue of their associated imagery and tactile experience, which affords facilitated explicit memory performance (Paivio, 1991).

One potential limitation of the present study was the relatively low memory accuracy rates. The average hit rates for both children and adults were in the mid 60s, suggesting that the recognition memory task was quite challenging. We presented participants with 120 words at encoding in order to test retrieval with a good number of items of each word type. The recognition memory task was unexpected; we did not warn participants that their memory would be tested because we wanted to assess incidental effects on memory. These methodological choices likely contributed to making the recognition memory task challenging for all participants and may have contributed to the different results we observed across hit rates and discrimination scores. Another limitation is created by the fact that we conducted the study in English. As such, our results cannot necessarily be generalized to other languages. In addition, the study was conducted with a WEIRD (Western, Educated, Industrialized, Rich, and Democratic) sample. Therefore, the findings cannot be applied to other populations and may not generalize to human conceptual development more broadly (Henrich et al., 2010; Simons et al., 2017). In future research, other populations and languages should be studied.

In conclusion, this study, for the first time, investigated the link between emotional enhancement of memory and word concreteness. The results showed that emotional valence plays an important role in lexical memory of both children and adults, particularly for abstract words. The results thus provide insights about how children acquire the meanings of abstract words. In keeping with the Affective Embodiment Account, and with multimodal accounts of lexical semantics, our findings point to emotion as a factor that shapes children’s word representations and helps them process and understand abstract concepts.

## Data Availability Statement

The datasets presented in this study can be found in online repositories. The names of the repository/repositories and accession number(s) can be found below: https://osf.io/69syt/.

## Ethics Statement

The studies involving human participants were reviewed and approved by University of Calgary Conjoint Faculties Research Ethics Board. Written informed consent to participate in this study was provided by the participants’ legal guardian/next of kin.

## Author Contributions

JK, DS, and PP conceptualized the study and jointly wrote the manuscript. JK tested the participants. JK and DS analyzed the data. All authors contributed to the article and approved the submitted version.

## Conflict of Interest

The authors declare that the research was conducted in the absence of any commercial or financial relationships that could be construed as a potential conflict of interest.

## Footnotes

1 Cohen’s *d* was calculated using this formula: 2 × t / √df_Error_.
